# Mechanistic Insights into the Metabolic Pathways and Neuroprotective Potential of Pentacyclic Triterpenoids: In-Depth Analysis of Betulin, Betulinic, and Ursolic Acids

**DOI:** 10.3390/biom16010025

**Published:** 2025-12-24

**Authors:** Mihai Adrian Socaciu, Zorita Diaconeasa, Dumitrita Rugina, Carmen Socaciu, Remus Moldovan, Simona Clichici

**Affiliations:** 1 Department of Radiology and Medical Imaging, Faculty of Medicine, University of Medicine and Pharmacy “Iuliu Hatieganu”, 400347 Cluj-Napoca, Romania; mihai.socaciu@umfcluj.ro; 2 Faculty of Food Science and Technology, University of Agricultural Sciences and Veterinary Medicine, 400372 Cluj-Napoca, Romania; zorita.sconta@usamvcluj.ro; 3 Faculty of Veterinary Medicine, University of Agricultural Sciences and Veterinary Medicine, 400372 Cluj-Napoca, Romania; dumitrita.rugina@usamvcluj.ro; 4 Department of Biotechnology, Research Centre for Applied Biotechnology in Diagnosis and Molecular Therapy, Biodiatech-Proplanta SRL, 400478 Cluj-Napoca, Romania; 5 Department of Physiology, Faculty of Medicine, University of Medicine and Pharmacy “Iuliu Hatieganu”, 400347 Cluj-Napoca, Romania; remus_ri@yahoo.com (R.M.) sclichici@umfcluj.ro (S.C.)

**Keywords:** pentacyclic triterpenoids, betulin, betulinic acid, ursolic acid, anticancer and chemoprevention, neuroprotection

## Abstract

Due to their complexity, both genotypic and phenotypic, neurodegenerative diseases are one of the main causes of death globally nowadays. Among phytochemicals of high scientific interest, based on experimental studies, pentacyclic triterpenoids (TTs), including mainly betulin, betulinic, and ursolic acid, became targets of scientific research in recent years, especially in terms of their biological activity and pharmacological potential. Due to their anti-inflammatory and antioxidant properties, as well as their involvement in cellular signal transductions, they have been observed to act as anticancer, chemopreventive, and neuroprotective agents. The aim of this review is to update the reader on the diversity, bioavailability, pharmacological properties, and neuroprotective effects of TTs, as biomolecules that can interfere with metabolic mechanisms related to neurodegeneration and restoring of neuronal integrity. Recent data were analyzed, with a particular focus on mechanistic insights related to their neuroprotective effects. Starting with their biosynthetic pathways, bioavailability, and involvement in specific metabolic pathways, their impact on neurological pathology and benefits as natural neuroprotection agents through specific signaling pathways are presented. Furthermore, emphasis will also be put on current challenges and future strategies that could develop TTs into effective compounds for neuroprotection and personalized medicine.

## 1. Introduction

Plants synthesize a large diversity of secondary metabolites, including terpenoids beside alkaloids, phenolics, and glucosinolates, responsible for multiple biological properties, defense against pathogens, and pharmacological activities [1,2,3,4]. Terpenoids are oxidized derivatives of terpenes, synthesized from five-carbon isoprene units (IPP) and include structures assembled in different ways, with high molecular diversity and complexity, and a wide range of biological and pharmacological properties and actions, including antioxidant, antiviral, antibacterial, antifungal, anti-inflammatory, and anticancer activities [3,4]. Pentacyclic triterpenoids (TTs) are derived from common precursors of triterpenes and steroids in the isoprenoid biosynthetic pathway. Many biosynthetic pathways of triterpenoids and saponins (glycosylated derivatives) and their metabolic regulation have been elucidated by multitopic technology [2]. Meanwhile, updated research data found these molecules may also exert neuroprotective effects, with their mechanisms of action, structure–activity relationships, and their involvement in the modulation of different intracellular pathways specific to neurological disorders being discovered [5,6,7,8].

The International Food Database (https://foodb.ca/ includes a list of the most representative pentacyclic triterpenoids, their classification (lupane, ursane, and oleanane groups). Besides the major TTs whose activity is detailed in this review, other TTs such as oleanolic, maslinic, asiatic, corosolic, and platanic acids, as well as their semi-synthetic derivatives, showed a large area of beneficial biological activities, as recently reviewed [9].

This review aims to synthesize recent data (mainly the last 7 years) related to the neuroprotective effects of the main TTs, betulin, betulinic acid, and ursolic acid, focusing on the mechanistic insights into the metabolic pathways and neuroprotective potential. Starting from their biosynthetic pathways and bioavailability, and specific pathways, their effects on neurological pathology and benefits as natural neuroprotection agents are presented.

## 2. Pentacyclic Triterpenoids: Biosynthesis and Bioavailability

Triterpenes’ biosynthesis begins at a genomic level, where terpene synthase gene families and the genes responsible for primary metabolism are expressed. Like other terpenoids, TTs are involved in transcriptional regulation and modulate epigenetic mechanisms and signaling pathways. They can affect gene expression by influencing histone modifications, DNA methylation, and microRNA (miRNA) levels, which in turn control the activity of Sp1 and Nrf2, as transcription factors. This regulatory role explains their involvement in cell proliferation, inflammation, and apoptosis. These highly functionalized molecules (C_30_ units) are produced through complex biosynthetic pathways, beginning with the MEP (methylerythritol phosphate) and MVA (mevalonic acid) precursors supplying the C_5_ units for the downstream [2,3,10,11,12,13,14].

**Figure 1:**
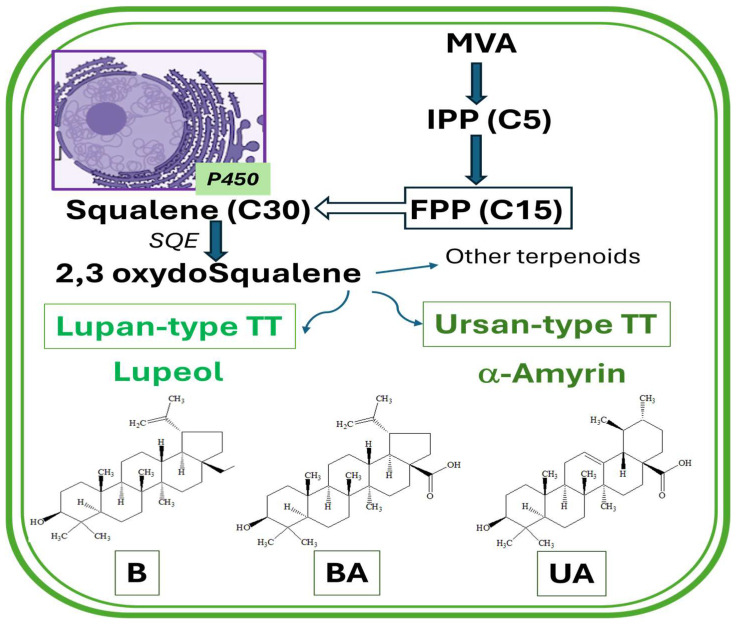
Schematic overview of TTs (C_30_) biosynthesis by the MVA pathway, directed to lupane- and ursan-types of pentacyclic triterpenoids (TT). MVA-mevalonic acid, IPP(C_5_)-Isopentenyl diphosphate, FPP(C_15_)-Farnesyl diphosphate, SQE-Squalene epoxidase, and P450-Cytochrome P450.

TTs are classified into six main classes, the most studied being the Lupane-type, which includes botulin (B) and betulinic acid (BA), and ursane-type T, which includes ursolic acid (UA), the focus of this paper [15,16]. Figure 1 presents a schematic overview of pentacyclic triterpenoid biosynthesis directed to lupane- and ursan-types.

At the primordial level, terpenoids are synthesized from IPP (C_5_ units) and released from the cytosolic mevalonic acid (MVA) pathway and the plastidial methylerythritol phosphate (MEP) pathway. IPP units represent the first common intermediate between the two pathways, while mono-(C_10_), di-(C_20_), and tetra-(C_40_) terpenoids are synthesized exclusively via the MEP pathway. Sesqui-(C_15_) and tri-(C_30_) terpenoids are created exclusively via the MVA pathway [1,10,12,13,14]. At the proteomic level, the proliferation of cytochrome P450 monooxygenases (P450) in the endothelial reticulum leads to accelerated metabolic processes, creating the structural and functional diversity of triterpenoids, from carboxyl and hydroxyl to carbonyl and epoxy derivatives [1,10,11,12]. The intersection point between these two major pathways in plants is embodied by 2,3-oxidosqualene, resulting from the activation of squalene epoxidase (SQE). Finally, their cyclization is performed via oxidosqualene cyclases (OSCs) and decoration via cytochrome P450 monooxygenases (CYP450s) and glycosyltransferases (GTs) [10,11,12,13,14,15].

Betulin (B) is the most abundant among TTs in nature and is especially found in plant species of the *Betulaceae* family (up to 40% among TTs), usually distributed in the birch outer bark. It is the precursor of betulinic acid (BA), naturally found in lower concentrations but with a greater potency and efficiency for drug development [15]. B acts as a precursor for BA; both compounds share similar pharmacological properties, like anti-inflammatory and anti-cancer effects by modulating key pathways such as NFκB and Nrf2 and inhibiting cyclooxygenase enzymes [17]. Ursolic acid (UA) is an isomer of BA, derived from α-amyrin, identified in the epicuticular waxes of fruit peels, as well as in herbs or spices like rosemary and thyme [4,17]. These molecules exhibit distinct and often complementary effects rather than synergistic interaction.

Table 1 includes the main resources of the other TTs, found in smaller concentrations in plants, but proving to also have beneficial activities.

Due to their low solubility in water, polar solvents, and aqueous environments, the utilization of their supramolecular architecture is restricted; therefore, their bioavailability is very low. Meanwhile, they showed unique self-assembly and co-assembly behaviors in relation to different physiological functions, and can construct bioactive delivery carriers due to their higher biodegradability, biocompatibility, and lower toxicity [27].

**Figure 2:**
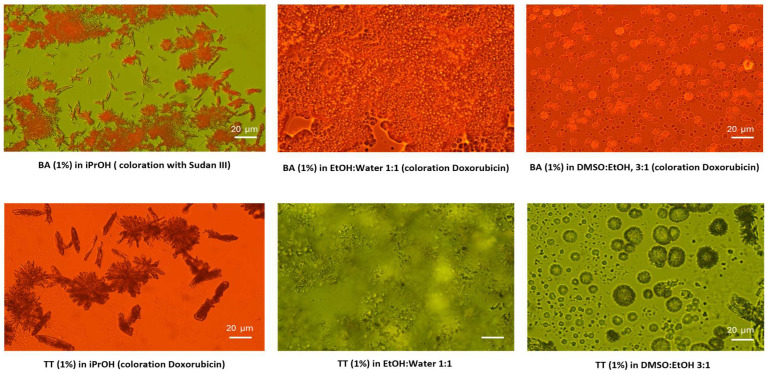
Optical Microscopy images of 1% pure betulinic acid (BA) and a 1% birch bark extract (TT) in different solvents: iso-propanol (iPrOH), ethanol (EtOH):water (1:1), and dimethyl sulfoxide (DMSO): EtOH, 3:1 [29].

Study of the self-assembly of several TTs, such as botulin and betulinic acid, as well as their derivatives from birch bark extracts, in different solvents has been reported [28,29]. Therefore, flowers and fibrillar networks can be converted into vesicles, spheres, or nanoemulsions of micrometer dimensions in the perspective of increasing their bioavailability. Recently, solvents such as ethanol, methanol, acetone, ethyl acetate, diethyl ether, chloroform, and dichloromethane were tested in the extraction process, and the dimensions of assemblies can range from nanoscale to several millimeters in length [4,28,29]. Figure 2 represents the different structures of 1% pure BA and a triterpenoid extract of birch bark (TT) containing a mix of lupeol, B, and BA, showing self-assembled crystals of acicular- or flower-type, or vesicles which form nanoemulsions in different solvents.

Different shapes and sizes were observed, depending on the solvent used. In iPrOH, acicular crystals and aggregated flower-like types with sizes from 1 to 40 µm were observed. The mix of terpenoids (TT extract of birch bark) showed a higher degree of crystallization, considering the dominance of betulin and the presence of lupeol in the extract. In EtOH:water (1:1), the crystals were partly dissolved, resulting in small-scale aggregates, while in DMSO:EtOH (3:1), vesicles with more or less homogeneous dimensions were formed: BA vesicles measured around 3–10 µm, while TT vesicles had more varied shapes and larger sizes, ranging from 1 to 20 µm.

The frequently used techniques of extraction are percolation, maceration, enfleurage, and Soxhlet extraction. Other techniques, such as supercritical CO_2_ extraction, microwave-assisted extraction, sonication, and pressurized liquids, were proposed as improved ways of high-quality extraction [30]. Over time, some reviews covered the literature data on the bioavailability of pentacyclic triterpenes ingested either from foods and medicinal plants or in their free form [30,31,32]. A promising protocol to increase B bioavailability in vivo for oral administration, which included solubilization in ethanol, mixing with olive oil or lard, followed by ethanol evaporation, resulting in an emulsion, was recently reported [33]. Since almost all TTs have poor solubility in aqueous media, which limits their study in vitro and for clinical use, for experimental trials, different organic solvents, such as ethanol, chloroform, DMSO, hexane, or dichloromethane, were used. As presented above, for many trials in vitro and in vivo [33,34,35], there were preferred mixes of DMSO with ethanol, where the homogeneity of the resulting vesicles was acceptable. According to our experience, as shown in Figure 2, the mix of DMSO and ethanol proved to have good solubilization and improved incorporation into lipid vehicles (liposomes or nanolipid complexes), resulting in bioavailable nanoformulations as reported recently [33,34,35,36]. Additionally, the encapsulation of birch callus cell extract in nanoliposomes to enhance the delivery and anti-cancer properties of B and BA was reported [37], and hybrid PLGA/lipid nanoparticles for UA delivery were reported [38].

## 3. Biological Activity and Pharmacological Potential

A recent review provided updated information about TT’s structural diversity, distribution, and pharmacological potential; their anti-inflammatory, anti-cancer, anti-diabetic, hepatoprotective, and neuroprotective activities were recently reported [39,40,41,42,43,44,45,46,47,48]. These effects were related to specific molecular mechanisms, including modulation of signaling pathways, enzyme inhibition, and interaction with cellular targets.

In-depth analysis was performed for lupeol, focusing on its multifaceted pharmacological properties, molecular pharmacology, and therapeutic advances, including anti-inflammatory, antioxidant, anti-cancer, and antibacterial effects, while considering also its poor solubility and bioavailability; therefore, nano-based delivery systems were also proposed in this case to enhance its bioavailability and amplify its bioactivity [40,41].

So far, most of the reports reviewed on the current data are focused on B and BA, but also UA. Specifically, their biological activity and pharmacological mechanisms were correlated with innovative strategies to enhance their clinical efficacy. A plethora of studies in vitro or in vivo showed, during the last decade, their pharmacological potential, including immune regulation, cardiovascular and hepatic protection, as well as anti-viral, anti-microbial, antioxidant, anti-diabetic, anti-inflammatory, anti-cancer and chemopreventive activities [39,42,43,44,45,46,47,48,49].

**Figure 3:**
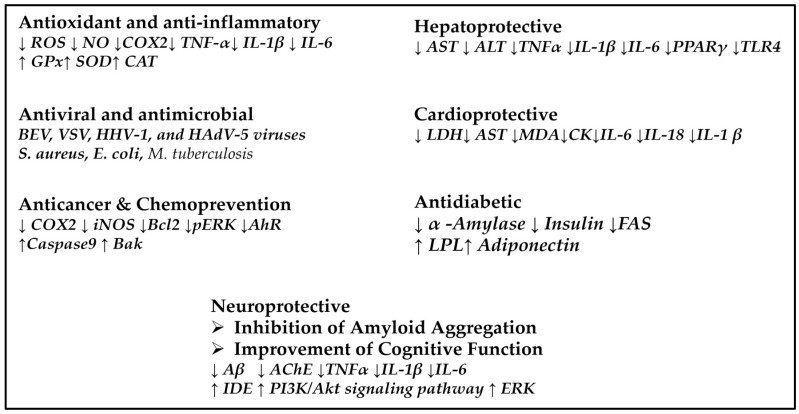
A general scheme reflecting the multiple biological properties of betulin, betulinic acid, and ursolic acid, and the specific biomarkers of metabolic pathways that are affected. The direction of arrows indicates increase or decrease of the biomarker levels.

Figure 3 summarizes the diverse biological activities related to their cytotoxicity and pharmacological potential, as reported by many scientific papers. As an addition to this diverse multi-potent activity, their neuroprotection by inhibition of amyloid aggregation and improvement of cognitive function is bringing special attention.

Many of these recent data are dedicated to the elucidation of mechanisms involved in the signal transductions at the cellular level, explaining their pharmacologic potential and for finding specific biomarkers selected as significant for their efficacy. According to Loboda [50], TTs and their derivatives block the expression of pro-inflammatory cytokines via NFκB, MAPKs, and Nrf2 pathways, as important regulators of the response to oxidative stress and inflammation in the body. BA suppresses STAT3 activation pathway (by phosphorylation via Janus kinases which include cytokines or IL-6 or EGF, bound to their receptors) in human multiple myeloma cells [51], while UA alleviated alcohol-induced liver injury via CASP3 in vivo [52] and also showed multiple benefits, as such or by its derivatives, as a potent pharmacological agent [47,53,54].

## 4. Mechanisms and Pathways Involved in Their Anti-Inflammatory and Anticancer Effects

Inflammation is often associated with the development and progression of cancer; therefore, targeting inflammation mechanisms represents an attractive strategy both for cancer prevention and therapy. Tumor-extrinsic inflammation is caused by many factors, including bacterial and viral infections, autoimmune diseases, obesity, and excessive alcohol consumption, all of which increase cancer risk and stimulate malignant progression. This section reviews the knowledge of the anti-inflammatory, anti-cancer, and chemotherapeutic effects of TTs, focusing on B, BA, and UA, evaluating their mechanisms in vitro and in vivo, through a variety of studies using different types of cancer cell lines and clinical studies.

Several studies have demonstrated that Nrf2 has a pivotal role in inflammation; it contributes to the anti-inflammatory process by recruiting inflammatory cells and regulating gene expression through the antioxidant response elements. In this context, the Nrf2-dependent anti-inflammatory phytochemicals become of high interest in drug discovery [55,56]. The anti-inflammatory effects of TTs have been identified since 1997 [57], but their therapeutic capabilities in inflammatory and immune processes were recently reported [58,59], in close relation with the anti-cancer and chemotherapeutic potential as reviewed recently [60,61]. As inflammation is a hallmark of many chronic diseases, B was found to decrease the levels of pro-inflammatory mediators, such as tumor necrosis factor (TNF), matrix metalloproteinases (MMP-2 and 9), and interleukins (IL-1, IL-2, IL-4, IL-5, IL-6, IL-13, and IL-17) [62].

Table 2 summarizes some recently published preclinical studies and clinical trials on the anti-inflammatory activity of some TTs.

TTs proved to inhibit cancer cell growth through mechanisms involving suppressed angiogenesis, selective apoptosis, and cell cycle arrest [69,70,71,72,73]. Induction of apoptosis is the primary anti-cancer activity of these compounds by affecting cancer cells differently, depending on cancer type, cell line, tumor size, source of betulin/betulinic acid, dose, treatment time, and the vehicle used for drug delivery.

A special focus was reported for lupane-TTs since 1995, when BA was identified as a promising anti-cancer drug that can induce apoptosis in melanoma cell lines in vitro and in vivo. Their effects were subsequently confirmed in a series of cancer cell lines, e.g., breast, colon, lung, and neuroblastoma. B, BA, and lupeol displayed a multitarget potential in anti-cancer strategies [74,75], while UA proved to induce apoptosis in a variety of human cell lines through specific pathways, e.g., inhibition of DNA replication, activation of caspases, inactivation of protein tyrosine kinases, and induction of Ca^2+^ release [76]. B and BA impact tumor cells mechanistically by primarily inducing apoptosis through mitochondrial damage and caspase pathway activation, and then, modulating factors like MAPKs and NFκB to reduce inflammation. UA reversely activates NF-κB, STAT3, and AKT, and thus inhibits the inflammatory networks, suppressing COX-2 and NO activity, promising anti-inflammatory agents for future TTs-based anti-cancer candidates [77].

Nevertheless, B and BA showed they have different targets and strengths. While B has shown strong efficacy against nervous system cancers like glioblastoma and neuroblastoma, BA had a broader-spectrum effect against melanoma, hepatic, and pancreatic cancer. BA was the most studied TT, considering its higher bioavailability and found to modulate various signal pathways and protein expressions associated with cancer growth, such as PI3K/AKT/mTOR, NFκB, JAK/STAT, p53, cyclin, and Cdk proteins, while inhibiting ROS production under oxidative stress conditions [78,79,80]. BA was shown to suppress the proliferation and migration of hepatoma cells, raise the ROS level, and increase the cellular oxidation level when ferrostatin-1 was used to induce the death of hepatoma cells by ferritinophagy and promote ferritinophagy-protein expressions [81]. The anti-cancer properties of BA are governed by mitochondrial signaling pathways and exhibit selectivity for cancerous tissue, leaving non-cancerous cells and normal tissue unaffected, especially in chemo-resistant cases [82]. To improve its bioavailability, different nano-delivery systems (polymeric or magnetic nanoparticles and conjugates, nanoemulsions, liposomes, nanosuspensions, carbon nanotubes, and cyclodextrin complexes were produced and showed enhanced effectiveness [34,35,82,83].

Not only melanoma cells proved to be strongly affected by TTs, as demonstrated by many experimental data [35,36,84,85], but also breast cancer cells [86,87,88], renal carcinoma [89], and gastrointestinal and colorectal cancers [90,91,92,93,94,95], with a special emphasis on inducing mitochondrial membrane dysfunctions.

A valid explanation of the impact of TTs and their differential effects on cells is performed by analyzing their distinct effects on membrane properties, e.g., fluidity, aggregation, and interaction with cholesterol. B and BA as well betulonic acid (a derivative of betulinic acid which contains a keto group instead OH at C3) have shown distinct effects: while B reduces membranes ‘fluidity and stimulates aggregation by its packing in the lipid bilayer, similarly to cholesterol, BA and betulonic acid increase fluidity, but simulates as well aggregation, having significant consequences on mitochondrial phosphorylation and production of H_2_O_2_. At high doses, it can induce membrane permeabilization [96]. Additionally, when a betulin conjugate with a penetrating cation F16 was applied, the potential of mitochondrial membrane was disturbed and induced superoxide overproduction in rat thymocytes and rat liver mitochondria in vitro, exceeding the effects of single B or F16, finally suppressing respiration and oxidative phosphorylation [97]. Also, bioactive functionalized conjugates of lupane-type TTs with triphenyl phosphonium and glucopyranosyl showed to improve targetability and cytotoxicity upon cancer cells, in comparison with the natural and glycosylated BA [98].

To notice also some contradictions in the literature regarding the efficacy of different TTs, which are explained by methodological differences in preclinical studies (origin and purity of TTs, different dose ranges, different carriers applied, purity and toxicity of solvents used to improve bioavailability).

Also, significant challenges need to be mentioned regarding the poor in vivo bioavailability of triterpenoids in animal models, whether administered orally or intravenously, making it difficult to compare and to establish standard dose–response relationships, a major barrier for their applicability in clinical practice. It is also obvious that it is still difficult to find the relevance of the in vitro concentrations used for in vivo conditions.

There are still a few data points to compare the comparative efficacy of B, BA, and UA with existing chemotherapeutic drugs. Instead, they can interfere with the mechanisms of action of existing drugs, e.g., UA and doxorubicin. While UA can induce apoptosis, its antioxidant activity can interfere with the therapeutic effect of doxorubicin, which induces the production of ROS. Considering the side effects, while conventional drugs often show long-term severe side effects, TTs are reported to have insignificant or absent toxicity in most studies, making them attractive candidates if their efficacy challenges can be overcome.

Current research focuses on developing semi-synthetic derivatives and advanced drug delivery systems (e.g., nanoparticles, liposomes) to improve the pharmacokinetic properties and target-specific delivery of TTs for clinical applications.

## 5. Toxicity Versus Cytotoxicity

One key aspect to evaluate the pharmacological potential of TTs is the balance between their toxicity on normal cells and tissues versus cytotoxicity against tumor cells and diverse pathological conditions. TTs are generally regarded as safe compounds with low toxicity profiles in preclinical studies (VI class of toxicity), with selective cytotoxicity towards cancer cells while having minimal or no harmful effects on normal, healthy cells. In a preliminary study, in 2008, a triterpene extract was tested in vitro, and an in vivo subchronic toxicity study by i.p. administration to rats and dogs was performed. Due to the poor aqueous solubility, the extract was suspended in sesame oil, and at 300 mg/kg, a maximum plasma concentration of 0.33 µg/m, betulin was detected after 28 daily applications. This toxicity study showed no toxicity and confirmed betulin bioavailability in oily suspension [44].

Later, the acute toxicity of B was studied on rats and mice as a single dose of 1000–16,000 mg/kg intragastric and in rats in a single dose of 250–4000 mg/kg i.p. No significant effects on body weight and no lethal effects were observed during 14 days after administration in rats and mice in all doses tested. LD_50_ was not achieved in all experiments. Skin irritation, edema, or infiltration at the injection site after intraperitoneal injection was not observed. Irritation of the gastrointestinal tract of rats and mice, and the location of all internal organs, was without pathology, and there were no significant changes in the mass coefficients of organs. Therefore, no toxic effects were observed after 14 days, and necropsy data confirmed the safety of B, being considered a non-toxic substance of VI class toxicity [99].

The primary challenge in the clinical application of these compounds is not toxicity but their poor water solubility and low bioavailability, which limit their absorption and effective concentration in the body. Research efforts focus on improving delivery methods (like liposomes or nanoparticles) rather than mitigating severe inherent toxicity, as reviewed recently [36]. Since B is poorly water-soluble and affects its pharmacokinetic behavior, possible localized tissue inflammation at the injection site may occur. Better delivery methods using bioavailable carriers to overcome their low bioavailability and enhance their potential therapeutic effects, and minimize toxicity were reported [90]. BA has also shown a similar profile, with low systemic toxicity but significant in vitro cytotoxic effects against tumor cells, and generally safe for normal cells.

Cytotoxicity studies employ cellular models and evaluate cellular line culture and measurement of parameters associated with cell proliferation, e.g., quantity, ability to divide, mitochondrial activity, condition of the cell membrane, or total content of DNA or proteins. Several stains are used to mark cytotoxic activity: evaluation of total protein content, MTT assay of mitochondria oxidoreductive activity, DAPI evaluation of total DNA content, and trypan blue staining of dead cells. The use of fluorescent stains makes it possible to apply flow cytometry as well as fluorescence microscopy to mark all: dead, living, and apoptotic cells [35]. BA showed selective, cytotoxic properties against tumor cells, with no activity toward normal cells of the body. MTT tests have shown cytotoxicity of BA in different cell lines of melanoma, ovarian carcinoma, lung cancer cells, and blood lymphoblasts. BA and UA derivatives (saponins) showed no cytotoxic activity toward human embryonic kidney cell derivatives and may be considered as potential, clinical, anti-cancer agents with selective cytotoxic activity [49].

Another study [100] evaluated the toxic potentials of BA and UA using rodent models (Swiss albino mice and Wistar rats) according to the OECD guidelines. Acute and 28-day sub-acute oral toxicity studies were conducted, and evaluations were made based on biochemical, hematological, and histopathological observations. Acute oral toxicity study revealed the oral LD50 of both the test compounds to be >2000 mg/kg in mice. Sub-acute administration of BA at 10 mg/kg body weight (b.w.) revealed a significant increase in serum glutamic oxaloacetic transaminase (SGOT), alkaline phosphatase (ALP), urea concentrations, and eosinophil and lymphocyte counts in rats. Animals administered with 10 mg/kg b.w. UA revealed elevated neutrophil count, SGOT, ALP, and urea concentrations, whereas white blood cells (WBC), lymphocytes, and platelet counts were found to be low. Histopathological examinations of body organs revealed alterations in the architecture of the liver, kidney, and spleen tissues. Notably, all these alterations were recoverable as evident in the satellite group, indicating a recovering pattern from the toxic effects caused by the oral administration of these phytocompounds. Although UA and BA possess several therapeutic properties, their long-term usage can cause low toxicity.

By treatments of human keratinocytes with BA, it was observed that cell senescence may be a response to parallel damage in the membranes of mitochondria and lysosomes [101]. By biochemical, immunocytochemical, and cytometric assays after challenging these cells with BA, the liposomal membrane leakage was noticed, leading to autophagy impairment, lipofuscinogenesis, genomic instability, and cell senescence. This work revealed that cell senescence’s initial trigger can be physical damage to membranes.

## 6. Neurodegeneration: General Mechanisms and Diseases

The brain is a complex and fragile living system, and brain-related diseases are caused by a progressive dysfunction, degeneration, and subsequent loss of neurons driven by oxidative stress and inflammation, and finally neuronal death. The diseases affecting the central nervous system (CNS) include neurodegenerative diseases like Huntington’s (HD), Alzheimer’s (AD), and Parkinson’s (PD), but also demyelinating diseases such as multiple sclerosis, cerebrovascular diseases like stroke, and infectious diseases like meningitis and encephalitis. Rare and fatal neurodegenerative disorders are the prion diseases in which misfolded proteins (prions) accumulate in the brain, causing nerve cell damage and symptoms like memory loss, dementia, and impaired mobility [102,103].

A healthy brain is protected by the blood–brain barrier (BBB), which is formed by the endothelial cells that line brain capillaries and support normal neuronal function by homeostasis of the brain microenvironment and restricting the pathogen and toxin entry to the brain. BBB dysfunction is implicated in many neurological diseases, from stroke, Alzheimer’s disease, to multiple sclerosis, and brain infections [104].

Microglia are the resident immune cells of the CNS, which exert modulating roles on neuronal synaptic development and function. Their role in BBB function, interactions with endothelial cells, astrocytes, and pericytes, were recently described as their involvement in the modulation of BBB function in neuroinflammatory conditions identified in stroke or neurodegenerative diseases. The potential of microglia to exert a dual role, either protective or detrimental, depending on disease stages and environmental conditioning factors, was also reported recently [105].

**Figure 4:**
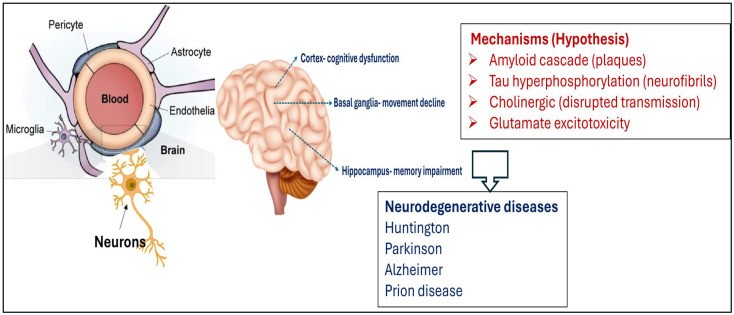
Representation of the BBB, the different localizations of brain dysfunctions identified in neurodegenerative diseases, and the main hypothesis related to mechanisms involved in such diseases.

Figure 4 shows the involvement of the blood–brain barrier (BBB), the different localizations of brain dysfunctions (in cortex, basal ganglia, and hippocampus) identified in NDDs, inducing cognitive dysfunction, movement decline, and memory impairment, respectively. The main hypothesis and related mechanisms leading to neurodegenerative diseases (HD, AF, PD, and prion) are also presented.

In recent years, the neurodegenerative disorders have become a major health concern and economic burden, with severe threats to human health, since they cannot be radically cured, instead relying mainly on drugs to alleviate their symptoms. Usually, these disorders turn worse over time due to the incapability of the neurons to self-regenerate, cell death or severe damage that occurs to the neural tissue, According to the World Health Organization statistics updated in 2024, the main and growing cause of morbidity, mortality, and disability (from 1990 to 2021) were neurological and psychiatric disorders, expressed in disability-adjusted life-years (DALY), affecting in average 3.40 billion individuals (43.1% of the global population) and the global DALY counts attributed to these conditions increased by 18.2% between 1990 and 2021 [106].

The central nervous system is a highly plastic network of cells that constantly adjusts its functions to environmental stimuli throughout life. Transcription-dependent mechanisms modify neuronal properties to respond to external stimuli, regulating numerous developmental functions, such as cell survival and differentiation, and physiological functions such as learning, memory, and circadian rhythmicity. The discovery and cloning of the cyclic adenosine monophosphate (cAMP) responsive element binding protein (CREB) constituted a big step toward deciphering the molecular mechanisms underlying neuronal plasticity [107].

Based on current knowledge focused on the molecular mechanisms involved in senescence and neurodegeneration, the main focus was related to AD and PD. Besides lifestyle, nutrients or phytochemicals may modulate age-associated molecular dysfunctions, their preventive or therapeutic benefits being emphasized. Several phytochemicals (mainly polyphenols, Sulfur organic compounds, and triterpenoids) have been shown to modulate the dysfunction of several key genes in the affected brain [107,108].

**Figure 5:**
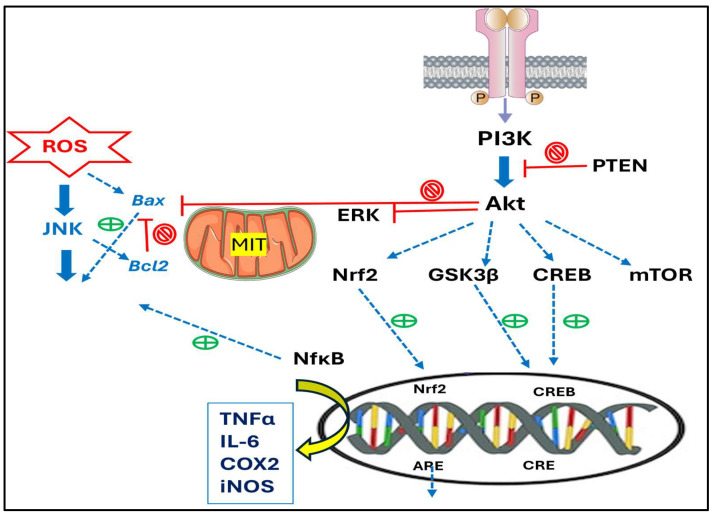
The PI3K/Akt pathway is related to neurodegeneration and its role in activating downstream effectors involved in cell metabolism, survival and apoptosis, differentiation, and proliferation. Their detailed meanings are presented in the abbreviation list.

For many of these activities, the essential mechanisms of action include PI3K/Akt, two key proteins for the intracellular signaling pathway. These proteins regulate cell growth, proliferation, and survival. PI3K is activated by growth factors and phosphorylates Akt, which triggers a cascade of downstream key events that control metabolism, gene expression, and cell cycle progression, with relevance in neurological disorders (as presented in Figure 5).

In the nervous system, the PI3K/Akt pathway contributes to neuronal growth, proliferation, and survival. Akt acts as a target for apoptosis and regulates cellular functions, e.g., leading to the nuclear translocation of Nrf2 and CREB, which promotes cell survival and prevents apoptosis. The loss or excessive activation of Akt determines the pathophysiological properties of a variety of complex diseases, including type 2 diabetes and cancer. Meanwhile, Akt is highly involved in the upregulation of the transcription factor Nrf2, which induces downregulation of the NFκB pathway, associated with anti-inflammatory and inhibition of oxidative stress. Another transcription factor, CREB, promotes the expression of neuroprotective proteins [49], responsible for brain development, growth, and differentiation. The downstream effector, GSK-3β, activated by Akt, inhibits the β-catenin expression and reduces neuronal activity. Moreover, Akt can also promote the mTOR expression, which regulates cellular nutrition and energy supply and stimulates protein synthesis. Akt can also inhibit the expression of the pro-apoptotic protein Bax and increase cell survival via the anti-apoptotic protein Bcl-2. Therefore, PI3K and Akt are activating downstream effectors involved in metabolism, cell survival and apoptosis, differentiation, and proliferation. The pathophysiological actions of TNF-α in neurodegeneration, e.g., in AD, play key roles as a potent pro-inflammatory and cytotoxic polypeptide in CNS disorders and brain trauma [109].

Mitochondrial quality control in neurodegeneration and cancer is a key factor and implies distinct therapeutic challenges considering their opposite outcomes in dysregulation: while in cancer, the specific mitochondrial pathways can be co-opted by cancer cells to enhance survival and resistance to therapy, in neurodegenerative diseases, a failure the mitochondrial dysfunction is irreversible by oxidative stress, and neuronal damage [110]. The therapeutic approaches to mitochondrial dysfunction are important, as shown for PD treatment [111]. The mitochondrial dysfunction leads to oxidative stress, damage to mitochondrial DNA, altered morphology, and ultimately neuronal death. Preclinical studies in animal models have shown the efficacy of mitochondrial-targeted antioxidants, while clinical trials, creatine and Coenzyme Q10 (CoQ10), which target energy metabolism, and triterpenoids were tested.

A transcriptional increase in the activity of the Nrf2/ARE pathway, which activates transcription of anti-inflammatory and antioxidant genes, producing neuroprotection, represents a promise for future therapeutic developments in the treatment of PD [110]. The diet influences, as a modulator, the inflammatory process in neurological diseases, e.g., the Mediterranean diet reduces inflammatory markers and oxidative stress, as well as influences the gut microbiome to reduce neuroinflammation [112].

For an accurate diagnosis and management of treatments, nowadays, new advanced digital neuroimaging techniques are available, to mention Magnetic Resonance Imaging (MRI) and Computer Tomography (CT) as well new functional imaging techniques use Tomography with Positron Emission (PET) which can identify structural changes (brain volume loss and atrophy, neurodegenerative changes from other causes of cognitive impairment and to assess vascular burden). Such techniques may detect pathological biomarkers, e.g., amyloid plaques in Alzheimer’s disease (AD) or dopamine transporter changes in Parkinson’s disease (PD), enhancing diagnostic accuracy and helping to track disease progression and treatment effectiveness [113,114,115].

To conclude, neurological pathology encompasses a wide range of disorders affecting the BBB, the brain, the spinal cord, and nerves, with a significant impact on an individual’s quality of life. The symptoms can range from cognitive decline, memory loss, and motor dysfunction to severe physical disability and psychological issues, but nowadays, new imaging techniques help with accurate diagnosis and monitoring treatments. Unfortunately, until now, such advanced techniques have not reported to evidence the effects of TTs, or to monitor the in vivo experiments.

## 7. Neuroprotection: Experimental Data and Mechanisms of Action

Neuroprotection refers to biological processes or interventions that preserve neuronal structure and function, slowing or eliminating destructive processes, and includes antioxidant, anti-inflammatory, and anti-apoptotic processes. The prevention and treatment of neurological disorders is challenging due to the complex and multifactorial mechanisms, including the permeability of the blood–brain barrier, limited regenerative capabilities, or the side effects and bioavailability of the drugs used. The scientific interest in natural compounds with neuroprotective potential increased significantly in the last 10 years, with many reviews being dedicated to natural resources and purified phytochemicals, especially terpenoids and polyphenols, e.g., resveratrol and curcumin [116,117]. The advances in the pharmacological activities of terpenoids and their impact on neurodegenerative disorders were recently reviewed [6,118,119,120,121].

The mechanisms involved in the neuroprotection via terpenoids were largely related to the modulation of PI3k/AKT/mTor signaling pathway [121], as presented in Figure 5. A special focus was dedicated to AD, terpenoids, and their glycosylated forms (saponins) with amphipathic properties [122,123,124,125,126], which showed improved therapeutic potential.

B was the most studied, having diverse pharmacological benefits and multi-target therapeutic potential. Considering its potential therapeutic effects and molecular mechanisms in AD, a recent study [124] determined specific molecular mechanisms using a network pharmacology (Pharm Mapper-assisted analysis) and experimental validation in vitro on HT22 cells to predict the target genes and databases for screening AD targets. The results of the molecular docking analysis revealed a strong binding affinity between B and the hub genes, and the enrichment analyses of GO and KEGG pathways indicated that its neuroprotective effects mainly involved the PI3K-Akt signaling pathway. The results of in vitro demonstrated that a 2 h pretreatment may ameliorate formaldehyde-induced cytotoxicity and decrease Tau hyperphosphorylation and ROS levels. Furthermore, the PI3K/AKT signaling pathway was activated, and the expression levels of downstream proteins, namely GSK3β, Bcl-2, and Bax, were modified. A comprehensive review of mechanistic studies about the neuroprotective properties of B, BA, and UA was reported last year [126,127].

The neuroprotective effect of terpenoids has gained attention in recent years due to the rising prevalence of neurodegenerative diseases and has been shown to exert neuroprotective effects through several mechanisms [120]. Studies regarding the therapeutic potential of lupeol, ursolic, and oleanolic acids in neurodegenerative diseases, neuropsychiatric diseases, and their mechanisms related to the inhibition of oxidative stress, neuroinflammation, and excitotoxicity were also reported recently [115,116]. Either pure molecules or extracts containing these three types of TTs showed similar mechanisms, from suppression of PI3K/AKT/mTOR expression, to inhibition of NO and attenuation of dopaminergic neurotoxicity [120,121,128,129,130,131,132,133,134]. Also, a number of synthetic drugs are prescribed to treat neurodegeneration, e.g., idebenone, diazepam, doxepin, and memantine hydrochloride, but all have unwanted side effects. Thus, developing natural neuroprotective agents with high efficacy and low toxicity may increase the therapeutic prospects for neurological disorders [110], as described below. Meanwhile, synthetic terpenoids showed high potential in experimental studies with a mouse model of PD [128].

To summarize, the main mechanisms involved in neuroprotection, as documented and mentioned above, are:

1. Anti-inflammatory activity. Since neuroinflammation and oxidative stress are central mechanisms to the pathogenesis of neurodegenerative diseases, at the molecular level, TTs exert anti-inflammatory effects mainly by inhibiting key pro-inflammatory pathways, including the NFκB pathway. This inhibition leads to the reduced expression of inflammatory cytokines like TNF-α and interleukins IL-1β and IL-6. Additionally, TTs activate antioxidant defense mechanisms by upregulating the expression of nuclear factor Nrf2, which enhances the transcription of antioxidant enzymes such as SOD and CAT. These actions reduce oxidative stress, considered a major contributor to neuronal damage and neurodegeneration, and therefore, TTs can modulate these pathways and can contribute to neuronal integrity and function.
2. Inhibition of amyloid aggregation. Since the accumulation of Aβ plaques is a hallmark of AD, at the molecular level, TTs proved to interact directly with Aβ peptides, preventing their misfolding and subsequent aggregation into toxic oligomers and fibrils. These compounds disrupt the β-sheet-rich structure of Aβ aggregates and enhance the activity of proteolytic enzymes to eliminate the Aβ peptides. This dual action not only prevents new plaque formation but also promotes the degradation and removal of existing plaques, reducing neurotoxicity and supporting neuronal health.
3. Improvement of Cognitive Function. First, TTs increase the synaptic plasticity via signaling pathways (PI3K/Akt and ERK), which are crucial for neuronal survival, growth, and connectivity. Secondly, TTs enhance the cholinergic function via inhibition of AChE and increase the availability of acetylcholine, a neurotransmitter vital for learning and memory. Collectively, these actions synergistically support the preservation and enhancement of cognitive abilities.

Considering betulin, betulinic acid, and ursolic acid, the most studied TTs, substantial neuroprotective effects through various mechanisms in both in vitro and vivo were identified in recent years. While preclinical studies show promise, few have progressed to advanced human clinical trials, highlighting the need for more human-based evidence to confirm safety and effectiveness.

Table 3 provides an overview of these effects, highlighting their molecular actions in different experimental settings. In vitro studies included mainly their anti-inflammatory and antioxidant activities, while in vivo studies assessed both the molecular and the overall impact on neurological health and function in animal models.

B, BA, and UA showed similar molecular mechanisms at the cellular level and exhibit significant anti-inflammatory and antioxidant properties, primarily through the inhibition of NFκB and the upregulation of antioxidant enzymes. They can also influence key signaling pathways such as PI3K/Akt or ERK, which are crucial for neuronal survival and function. That is the reason why TTs are so versatile, being able to be considered biomolecules with high potential in neuroprotection and treatment of neurological diseases. Nevertheless, B can protect better against ER stress and neuron degeneration, being suitable for use in PD, while BA and UA can be used in AD by inhibiting amyloid-beta aggregation, enhancing the cognitive functions, and promoting neuronal growth and survival. BA’s stronger apoptotic and neurotrophic effects, along with UA’s anti-amyloid and cognitive-enhancing properties, differentiate them from each other and from B [136,139,146].

**Figure 6:**
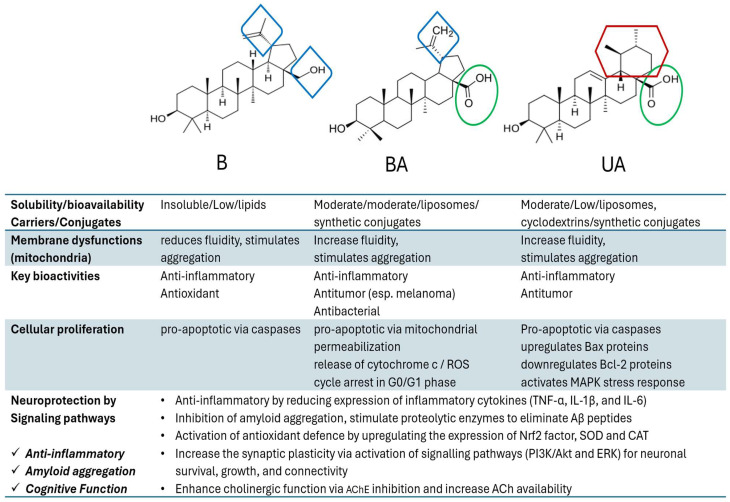
Summary of key mechanisms induced commonly or differentially by the three TTs analyzed in this review.

Figure 6 includes a summary of the key aspects related to the pharmacological activity of B, BA and UA, and especially their involvement in neuroprotection via interference with cellular signaling.

In its upper part, the figure shows the structural differences between the three TTs (at the C28 position and in the skeleton part or functional groups) underlying the relationship between their chemical structures, polarity, solubility, and bioavailability. These properties represent a key problem to be solved in experimental studies, since a large variability of results was dependent on these molecules’ presentation to membranes and cells, targeting metabolic dysfunctions. In general, experimental data showed increased efficacy of BA vs. B and BA vs. UA, but after appropriate solubilization. To increase accessibility and efficiency upon cellular proliferation and apoptosis or neuroprotection, appropriate carriers (lipids, liposomes, nanoparticles) or conjugates (natural or semi-synthetic) proved to be more beneficial, inducing similar mechanisms of action (as shown above), and attenuating the structural differences between these molecules.

## 8. Challenges and Future Directions

Significant neuroprotective properties of B, BA, and UA were reported in the last decade. They are generally regarded as having low toxicity at therapeutic doses. However, like all bioactive compounds, their safety profiles are dose-dependent and related to their solubility and bioavailability. An important disadvantage of B, BA, and UA is their low bioavailability due to their poor water solubility and limited absorption in the digestive tract. Recent research on innovative extraction methods and the use of compatible vehicles for delivery is yet to be available, and encouraging to find improved (nano)formulations to be more efficient as therapeutic compounds in neuroprotection.

When used within therapeutic ranges, TTs have the potential to be safe and effective neuroprotective agents. Considering the dose-dependence, and the adverse effects observed at high doses a careful dosage management and further toxicological studies are needed to obtain information on the tolerable doses and potential adverse effects.

Among the major neurodegenerative disorders, AD and PD are a huge socio-economic burden. Many medicinal plants and their secondary metabolites (e.g., ginseng, *Gingko biloba*, *Curcuma longa*, *Bacopa monnieri*, *Withania somnifera*, *Camellia sinensis*), have been known for their neuroprotective properties, terpenoids being one of the most noteworthy and extensively researched categories. These are reported with the ability to alleviate symptoms, the major mechanisms identified for phytochemicals to exert their neuroprotective effects and maintenance of neurological health in aging, include antioxidant, anti-inflammatory, antithrombotic, antiapoptotic, AChE, and monoamine oxidase inhibition and neurotrophic activities. The mechanisms of action of some of the major herbal products from different regional sources, according to their molecular targets, were recently reported, as presented above.

However, despite the considerable progress made in elucidating their neuroprotective mechanisms, several challenges and unanswered questions remain. These include the need for further elucidation of precise mechanisms of action of triterpenoids in neuroprotection, the structure-activity relationships of different triterpenoids, and optimization of delivery methods to enhance their bioavailability and brain penetration.

Future studies. Although many studies have investigated the mechanisms underlying their neuroprotective activities, the precise mechanisms are still unknown, particularly at the cellular and molecular levels, suggesting that machine learning (ML)—AI-based techniques can be employed for this purpose in the near future, using omics-derived large datasets of TTs structures correlated with their biological activities. Furthermore, high-throughput techniques like AI-powered metabolomics, pharmacogenomics, microbiome analysis, and genetic profiling can be used to maximize the utilization of triterpenoids in neuroprotection. Moreover, AI-powered metabolomics, pharmacogenomics, microbiome analysis, and genetic profiling can be used to optimize the use of triterpenoids in personalized medicine.

One major future area should focus on exploring possible synergistic effects of triterpenoids with other phytochemicals to provide enhanced neuroprotective outcomes and to tailor their use for personalized medicine. Advancements in molecular imaging, with new radiotracers and improved resolution, hold the potential to more accurately diagnose conditions based on both structural and molecular changes, potentially improving the evaluation of neuroprotective agents in the future.

## 9. Conclusions

The general focus of the scientific literature regarding the benefits of pentacyclic terpenoids is on their antioxidant and anti-inflammatory activities, and recently, more on their neuroprotective potential. Regarding the mechanistic insights, the regulation of different metabolic pathways, especially activation of the PI3K/Akt pathways, could lead to a more comprehensive understanding of their action, such as reduction in oxidative stress, downregulation of the expression of pro-inflammatory cytokines, which protect neurons more effectively. While direct evidence on the synergistic effects of betulin, betulinic acid, and ursolic acid specifically is still emerging, the complementary nature of their mechanisms of action provides a strong rationale for their combined use. Also, their combined effect might result in a more robust suppression of inflammatory processes, thereby providing stronger neuroprotection. Future research focusing on their combined neuroprotective effects could reveal significant therapeutic potential, offering more effective strategies for the prevention and treatment of neurodegenerative diseases.

Collectively, TTs offer a compelling combination of benefits, making them strong candidates for the development of new therapies aimed at preventing and treating neurodegenerative diseases. Future research focused on optimizing their bioavailability and understanding their synergistic effects could significantly advance their application in clinical settings, providing new hope for patients suffering from debilitating neurological conditions.

Additionally, the development of novel delivery systems to improve the bioavailability and penetration of the central nervous system could facilitate the transition of TT studies from preclinical into clinical trials and could enhance their efficacy in clinical applications.

## Figures and Tables

**Table 1:** Details about the main sources of oleanolic, maslinic, asiatic, corosolic, and platanic acid, their biological activities, and relevant sources of information, including their codes in the Food Database (FDB).

Name (TTs Group)	Main Plant Resources	Biological Activities	Ref.
Oleanolic acid (oleanane group) FDB013034 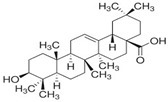	Oleaceae family (mainly olive) Cranberry, cloves Thyme, sage	Anti-inflammatory, anti-tumor, hepatoprotective Anti-diabetic, anti-hypertensive Anti-microbial, anti-parasitic	[9,18,19,20]
Maslinic acid (oleanane group) FDB013041 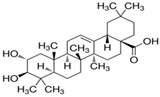	Virgin olive oil Hawthorn, pomegranate Eggplant, spinach, mustard	Anti-viral, Anti-fungal, Anti-bacterial Antioxidant Anti-diabetic, Anti-inflammatory Cardio protective, Neuroprotection	[9,18,19,21,22,23]
Asiatic acid (ursane group) FDB014909 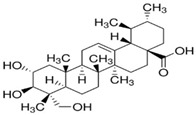	Edible and medicinal plants, e.g., centella asiatica, Guava/pomegranate	Stimulates collagen production Wound healing, anti-diabetic, Neuroprotective, cardioprotective, anti-microbial, anti-tumor	[9,18,19,24]
Corosolic acid (ursane group) FDB013735 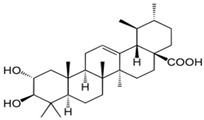	Banaba (*Lagerstroemia speciosa*) from tropical areas	Reduction in the gluconeogenesis, impairment of starch and sucrose hydrolysis, and enhancement of the cellular uptake of glucose	[9,10,19,25]
Platanic acid (nor lupane) No FDB-ID 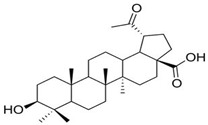	Low content in sycamore trees (*Platanus* sp.), *Melaleuca leucadendra* Obtained by partial synthesis from B or BA	Used as a scaffold for the synthesis of cytotoxic derivatives (amines, amides, and oximes) and their screening for cytotoxicity	[9,26]

**Table 2:** Mechanisms and key molecules involved in the anti-inflammatory activity of lupane-tts, botulin, and betulinic acid.

Molecules Studied	Mechanisms and Effects	References
Betulin and derivatives	Selective inhibition of TNF, MMPs, iNOS expression, NO inhibition, and suppression of the expression of interleukins	[62]
Betulin	Reduced inflammation in mouse chondrocytes, amelioration of osteoarthritis via AKT/Nrf2/HO-1/NF-B axis	[63]
Betulin	Modulator of the Glucocorticoid Receptor	[64]
Betulin	Inhibition of pro-inflammatory cytokines via STAT3 signaling in human cardiac cells	[65]
Betulin-NLC-hydrogel	Skin anti-psoriatic activity, enhanced skin hydration and lipid restoration, and reduction in cytokine levels	[66]
Betulinic acid	Antioxidant, anti-inflammatory, and anti-AChE activity of betulinic acid and 3β-acetoxybetulinic acid from *Melaleuca bracteata*	[67]
Lupane-TTs (*Maytenus* sp.)	Inhibition of NO and PGE2	[68]

**Table 3:** Overview of the recent in vitro and in vivo studies regarding the neuroprotection activities of betulin, betulinic acid and ursolic acid. ↓ inhibition/suppression/reduction, ↑ enhancement/increasing.

TTs	Cell Line/Animal Model	Concentration	Mechanism of Action	Biochemical Markers	Ref
In vitro studies					
Betulin	Neuronal hippocampal HT22 cell line	10 µM	antioxidant activity reduced cellular damage protection from ER stress increase HO-1 expression	↓ ROS ↓ Caspase12 genes ↑ HO-1 genes ↓ CHOP genes	[135]
	microglial cell BV2—LPS induced neuroinflammation	250 μg/mL	reduction in iNOS expression cytokines’ inhibition downregulated NFκB/p65 phosphorylation	↓ NO production ↓ NfκB ↓ TNFα, IL6, IL1β	[136]
	differentiated SH-SY5Y neuroblastoma cells	1–30 µM	protective effects against H_2_O_2_-induced oxidative stress Inhibition of apoptosis	↓ ROS ↓ apoptotic cells vs. H_2_O_2_-treated cells	[137]
	9 human neuroblastoma cell lines	0–20 µg/mL, 6 days	morphological changes in 3 days. Reduced axonic-like extensions, non-adherent, and condensed cells typical of apoptosis DNA fragmentation (ladder formation in the 100–1200 bp region in neuroblastoma cells	↑ apoptosis ↑ DNA fragments	[138]
	neuroectodermal tumor cells (neuroblastoma, medulloblastoma, glioblastoma and Ewing sarcoma cells)		direct effect on mitochondria, independent of p53 protein accumulation death-inducing ligand/receptor systems such as CD95 mitochondrial perturbations antitumor activity on neuroblastoma cells resistant to CD95/on doxorubicin-triggered apoptosis	↑ apoptosis ↑ cytochrome c ↑ caspases ↑ Bcl-2 ↓ proliferation	[139]
	brain tissue homogenates treated ex vivo with 0.1 mM FeSO_4_ for 30 min at 37 °C	10µM	pro-apoptotic effect reversal of suppressed levels of GSH, SOD, CAT, and ectonucleotidase activities. induction of oxidative neurotoxicity Bypass resistance to apoptosis-inducing agents (CD95 or doxorubicin).	↓ MDA ↓ NO ↓ ATPase ↓ AcChol ↓ α-chymotrypsin ↓ seleno- metabolism ↓ PI-signaling	[140]
Betulinic acid	BV2 microglial cells	10 µM	suppress M1 phenotype expression promote microglia M2 polarization anti-neuroinflammatory effects via CaMKKβ-dependent AMPK activation.	↓ TNFα release ↑ IL10 release ↓ IL6 ↓ IL1β ↓ mRNA ↓ iNOS ↓ CD16 ↓ CD68	[141]
	ZIKV infected Neural Cells	50 µM	antiviral activity via PI3K/Akt signaling pathway	↑ PI3K/Akt signaling pathway	[142]
	HD models in vitro: HEK-293T and NIH 3T3 cell lines In vivo: C57BL/6 male mice	BAH 5–20 µM In vivo (30 mg/kg)	inhibition of HIF and PHD2 HIF activation Action via protein phosphatase 2A (PP2A). Reduced striatal neurodegeneration	↓ PHD2 phosphorylation ↑ HIF-1α activation stability	[143]
Ursolic acid	PC12 nervous cells with Aβ_25–35_ induced toxicity	20 µM	anti-inflammatory effect nuclear translocation of the p65 subunit of NFκB inhibition of proteins’ phosphorylation	↓ iNOS ↓ COX2 ↓ IκBα ↓ ERK1/2 phosphorylation ↓ p38 ↓ JNK phosphorylation	[144]
	PC12 cell line	50 and 125 µM	antioxidant activity attenuate DNA fragmentation attenuate Aβinduced apoptosis	↓ ROS ↓ caspase3	[145]
In vivo studies					
Betulin	transgenic *Caenorhabditis elegans* (roundworm nematode) as models of PD	0.5 mM	Anti-PD activity: protection from 6-hydroxydopamine degeneration decreased inflammation increased life span downregulation of the apoptosis gene pathway egl-1 enhancement of proteasome activity by promoting Rpn1 expression	↓ αsyn nuclei ↓ Monocyte chemotactic protein1 ↓ PGsynthase2 ↓ iNOS ↓ egl-1 genes	[146]
	AD model Wistar albino male rats with okadaic acid	20 mg/kg/day, i.p).	Regulation of adipokine gene expression and iron accumulation, reduced hippocampal oxidative metabolism	↑ Antioxidant ↓ iron accumulation	[147]
	Rats with Diabetus induced with streptozotocin (30 mg/kg, ip).	20 mg/kg, 40 mg/kg	restored SOD activity upregulation of Nrf2, HO1 expression protective effect on cognitive decline through the HO1/Nrf2/NFκB pathway improved glucose intolerance and learning performance	↓ cytokines ↓ MDA ↓ cytokines ↓ IκB, NFκB phosphorylations	[148]
Betulinic acid	Wistar rats with AD induced with Aβ (0.1 μM/5 μlPBS/rat	0.2 and 0.4 μM/10 μL DMSO/rat (i.h.)	restored memory and anxiety, anxiolytic and antidepressant effects prevention of AD-induced neurobehavior prevention of LTP deficits at a molar ratio of 1:4 (Aβ:BA).	↑ proteasome ↑ hippocampal potentiation ↑ LTP parameters	[149]
	Induced stroke Wistar rats model (Middle cerebral artery occlusion) i.v.adm with AMD3100, for delivery of NA1	1 mg BAM	targeted BA release in acidic ischemic tissue improved recovery from stroke efficacy enhanced by encapsulated NA1 increased survival	Enhance the efficacy of the neuroprotective peptide NA1	[150]
	Wistar Rats with oxygen and glucose deprivation to induce neuronal injury	Pretreatment with BA	Attenuation of hippocampal neuronal injury, up-regulation of Bcl-2, downregulation of Bax, inactivation of caspase-3	↓ MDA ↓ ROS ↓ Bax ↑ Bcl-2 ↓ Caspase-3	[151]
	Wistar rats as a PD model induced with LPS and FeSO_4_.	BA 10 mg/kg	reversed behavioral deficits mitigated immunohistopathological and biochemical abnormalities reduce ferroptosis, and apoptosis biomarkers implicated in neurodegeneration	↑ TyrOHase ↓ α-syn ↓ SOD	[152]
	Wistar rats with vascular dementia induced by carotid artery occlusion	Oral, 10 and 15 mg/kg/day for 1 week	neuroprotective effect in a dose-dependent manner re-established cerebral blood flow, restored behavioral parameters fewer pathological abnormalities reduced inflammatory parameters. decrease in microgliosis	↑ cAMP, cGMP ↓ inflamation ↓ oxidative stress	[153]
	Glioblastoma cells and intracranial xenograft glioblastoma mouse models.	Nanoemulsion of BA in DMSO, Ethyl acetate, and polyvinyl acid 0–15 µg/mL	suppression of glioma cell proliferation, arrest the cell cycle in the G0/G1 phase. Downregulated Akt/NFκB-p65 signaling pathway cross the BBB and increase the survival time, anti-tumor effect	↑ apoptosis ↑ CB1/CB2 receptors ↓ Akt/NfκB-p65.	[154]
Ursolic acid	Mice model of Traumatic Brain Injury	100, 150 mg/kg	antioxidative and anti-inflammatory effects reduce brain oedema reduced neurological insufficiencies	↑ Nrf2 ↑ pAKT ↓ MDA ↑ SOD ↑ GPx	[155]
	Rats model of subarachnoid hemorrhage brain injury	50 mg/kg	antioxidative and anti-inflammatory effects: inhibition of apoptosis improved neuronal survival inhibition of caspases 3 and 9	↓ ICAM ↓ NFκB ↓ IL1β, IL6 ↓ TNF α ↓ TLR4 ↓ iNOS ↓ MMP9 ↓ MDA, SOD, CAT ↑ GSH/GSSG ratio	[156,157]
	Mice model of spinal cord injury	25, 50 mg/kg	neuro regeneration recover motor functions recover axonal regrowth decrease astrogliosis	↓ IL6 ↓ TNF α	[158]
	Nrf2 and wildtype mice	130 mg/kg	improved neurological deficit in acute stroke reduce infarct volume prevent ischemic damage antioxidant and anti-inflammatory responses	↑ Nrf2 mRNA ↑ HO1 mRNA ↓ TLR4 ↓ NFkB	[159]
	Rat PD model established by rotenone infusions	30-day Adm of 5, 10 mg/kg	improved mitochondrial enzymatic activity and MtCO1 gene expression	↑ CAT, ↑ SOD, ↑ GSH, ↓ MDA ↓ TNF-α, ↑ TyrOHase positive neurons	[160]
	PD Wistar rat model	UA-THP i.v. 25 mg/kg for 21 d	Activation of PP2A/PHD2/HIF pathway Reduction in oxidative stress Stimulation of TyrOHase-positive neurons Prevent neuronal loss Decrease in reactive astrogliosis and microglial activation	↓ glial fibrillary protein ↑ CAT,↑ GSH, ↓ MDA ↓ NF-κB, ↓ TNF-α, ↓ IFN- ↓ IL-12, ↑ IL-4, -10	[161]

## Data Availability

The original contributions presented in this study are included in the article. Further inquiries can be directed to the corresponding author(s).

## Abbreviations

The following abbreviations are used in this manuscript:

Abbreviation	Name
A-Syn	Alpha-Synuclein
Ache	Acetylcholinesterase
AD	Alzheimer Disease
Aβ	Amyloid-Beta
Akt	Protein Kinase B
AMD3100	A Chemokine Receptor CXCR4 Antagonist
B	Betulin
BA	Betulinic Acid
BAH	Betulinic Acid Hydroxamate
BAM	Betulinic Amine
BAX	Bcl2- Associated X
Bcl-2	B-Cell Lymphoma 2
CAT	Catalase
CREB	cAMP-Response Element Binding Protein
CT	Computer Tomogaphy
ER	Endoplasmic Reticulum
FT	Frontotemporal Dementia
Gpx	Glutathione Peroxidase
GSH	Glutathione
GSK-3β	Glycogen Synthase Kinase-3 Beta
GSSG	Glutathione Disulfide
HIF	Hypoxia-Nducing Factor
ICAM- 1	Intercellular Adhesion Molecule-1
IDE	Insulin-Degrading Enzyme
IL1β	Interleukin -1β
IL-6	Interleukin-6
I.H.	Intra-Hippocampal
I.V.	Intra-Venous
Inos	Inducible Nitric Oxide Synthase
LPS	Lipopolysaccharide
NO	Nitric Oxide
HD	Huntington’s Disease
IPP	Isopentenyl Diphosphate
MAPK	Mitogen-Activated Protein Kinase
MDA	Malondialdehyde
MEP	Methylerythritol Phosphate
MIT	Mitochondria
MMP	Matrix Metalloproteinase-
Mtco1	Mitochondria Cytochrome Oxidase
MRI	Magnetic Resonance Imaging
MVA	Cytosolic Mevalonic Acid
Mtor	Mammalian Target of Rapamycin
NF-Κb	Nuclear Factor-Κb
Nrf2	Erythroid 2-Related Factor
OECD	Organization for Economic Cooperation and Development
Oscs	Oxidosqualene Cyclases
P450s	Cytochrome P450 Monooxygenases
PBS	Phosphate-Buffer Saline
PD	Parkinson Disease
PET	Tomography with Positron Emission
PGE2	Prostaglandin E2
PI	Phosphatydyl Inositol
Phds	Prolyl-Hydroxylases
PP2A	Phosphatase 2A
PI3K	Phosphoinositide 3-Kinase
ROS	Reactive Oxygen Species
Shcs	Squalene-Hopene Cyclases
SOD	Superoxide Dismutase
TBI	Traumatic Brain Injury
TLR4	Toll-Like Receptor 4
TNF-α	Tumor Necrosis Factor-A
TyrOHase	Tyrosine Hydroxylase
UA	Ursolic Acid
UA-THP	Ursolic Acid Tetrahydroyridine Derivative
